# Artistic and Playful Resources as Mental Health Support in the Life Trajectories of Trans and Gender-Diverse People: A Qualitative Study from a Public Health Perspective

**DOI:** 10.3390/ijerph23030341

**Published:** 2026-03-09

**Authors:** Eduardo Name Risk, Jhully Cristine Ananias Boaro

**Affiliations:** Departamento de Psicologia, Centro de Educação e Ciências Humanas, Universidade Federal de São Carlos, São Carlos 13565-905, SP, Brazil; jhullyboaro@estudante.ufscar.br

**Keywords:** trans and gender-diverse populations, mental health inequalities, social determinants of health, qualitative research, art-based practices, public health

## Abstract

**Highlights:**

**Public health relevance—How does this work relate to a public health issue?**

- Trans and gender-diverse mental health inequalities are associated with structural stigma and restricted access to affirmative health services.
- Non-biomedical practices operate as informal mental health resources across trans and gender-diverse life trajectories.

**Public health significance—Why is this work of significance to public health?**

- The study contributes to public health by providing qualitative evidence of mental health support mechanisms beyond biomedical and clinical frameworks.
- It advances life-course and social determinants approaches to trans and gender-diverse mental health using qualitative evidence.

**Public health implications—What are the key implications or messages for practitioners, policy makers and/or researchers in public health?**

- Mental health policies may integrate non-biomedical and culturally situated practices as complementary care strategies.
- Public health research should incorporate everyday and community-based resources in trans and gender-diverse mental health models.

**Abstract:**

Trans and gender-diverse (TGD) people experience significant mental health inequalities. These disparities are associated with structural stigma, social exclusion, and persistent barriers to accessing health services, representing a critical public health issue. Although existing research has largely emphasized biomedical and clinical responses, less is known about non-biomedical resources that support mental health across life trajectories. This qualitative study explored, based on participants’ narratives, how artistic and playful resources contribute to mental health across life trajectories, considering contexts of social inequities and social determinants of health. Four trans and gender-diverse participants aged 18–27 years were recruited through snowball sampling and took part in two in-depth semi-structured interviews conducted remotely. Data were analyzed using Reflexive Thematic Analysis combining deductive and inductive coding. Findings indicate that artistic and playful practices were described as non-biomedical resources for emotional regulation, coping with minority stress, identity affirmation, and social connection across different life stages. These practices were narrated as helping participants manage psychological distress associated with stigma, discrimination, and limited access to affirmative mental health care. From a public health perspective, the results underscore the importance of recognizing culturally situated, everyday expressive practices as complementary forms of mental health support. Integrating such resources into broader mental health strategies may contribute to more comprehensive, equitable, and non-pathologizing public mental health approaches for trans and gender-diverse populations.

## 1. Introduction

In Brazil, within the scope of the Brazilian Unified Health System (Sistema Único de Saúde—SUS), the Transsexualization Process consists of a set of guidelines aimed at the provision of comprehensive health care for transgender, transsexual, travesti, and nonbinary individuals who seek bodily modifications through the alignment of primary and secondary sex characteristics with their gender identity [1]. In Brazil and other Latin American contexts, “travesti” designates an identity category historically constituted within social movements and marked by specific social and political meanings. Travestis in Brazil have been disproportionately exposed to stigmatization, violence, and social exclusion, often intersecting with racial and socioeconomic inequalities. In some contexts, “trans woman” has been adopted as a more institutionally legible category, which may obscure the specific historical and political meanings of travesti as a form of self-identification and a site of dispute and recognition. For this reason, “travesti” is not treated here as a simple synonym for “trans woman,” but as a culturally situated category relevant to public health inequities and access to care [2,3].

Specifically, Ordinance No. 2/2017, which consolidates national health policies within the SUS, expanded and redefined the Transsexualization Process. This policy establishes that health care for transgender, travesti, and nonbinary people should not be limited to medical treatments, surgical procedures, or bodily interventions, but must involve multiprofessional care. In accordance with the principle of comprehensiveness that underpins the SUS, Primary Health Care is defined as the main entry point for the transgender population and should provide welcoming, humanized, and discrimination-free care by both health professionals and service users. These principles apply not only to Primary Health Care, but also to all levels of care and their respective services. Ambulatory care includes clinical follow-up, pre- and postoperative care, and hormone therapy [4].

In 2023, the Specialized Health Care Secretariat of the Brazilian Ministry of Health established a working group to revise the Transsexualization Process. These efforts resulted in the proposal of the Specialized Health Care Program for the Trans Population, which is expected to be incorporated into the National Policy on Specialized Health Care. However, as the ordinance regulating this program has not yet been published, the Transsexualization Process remains in effect. Once implemented, the program aims to ensure comprehensive health care for transgender, travesti, and nonbinary individuals throughout the life course, highlighting the need to reduce access barriers across different levels of health care [5].

Despite these advances, transgender, travesti, and nonbinary individuals in Brazil continue to face substantial barriers to both mental and physical health care, compounded by social inequities [6]. In this study, we use the term trans and gender-diverse (TGD) to refer to individuals who identify as transgender and/or nonbinary, including travestis in the Brazilian context. For readability, the term “trans” is subsequently used as shorthand for trans and gender-diverse (TGD), unless otherwise specified.

Although definitions vary, social determinants of health broadly refer to the living and working conditions of individuals and population groups and their relationship with health outcomes. These determinants include social, economic, cultural, ethnic/racial, psychological, and behavioral factors that may lead to health problems or increased health risks for specific populations [7]. Accordingly, the social determinants of health framework explains how health outcomes are influenced not only by individual factors but also by broader social and economic conditions, such as income, housing, and discrimination, which directly affect quality of life and health status [8].

Globally, the TGD population faces significant barriers to accessing health care, constituting a major public health concern. A scoping review based on 45 international studies identified key obstacles to health care access for trans people, including limited provider knowledge of transgender health, long waiting times, high treatment costs, low social support, and pressure to conform to binary gender identities [9].

Trans and gender-diverse (TGD) people are consistently exposed to discrimination and social rejection, which are central components of minority stress and are associated with increased mental health risks [10,11,12]. Minority stress includes both distal stressors—such as structural discrimination, harassment, and violence—and proximal stressors, including anticipated rejection, internalized stigma, and heightened vigilance in social contexts [10]. These chronic stress exposures are associated with higher prevalence of depression, anxiety, psychological distress, and suicidal ideation among trans and gender-diverse populations, with rates substantially exceeding those observed in the general population [11,12,13]. Empirical evidence further demonstrates that exposure to identity-based discrimination and conversion efforts is significantly associated with increased psychological distress and suicide attempts [14]. From a public mental health and social determinants perspective, these disparities should not be understood as intrinsic to gender diversity, but rather as consequences of systemic social exclusion and structural inequities [8,11].

In Brazil, a qualitative study conducted with 52 trans women and *travestis* (mean age: 35 years) reported widespread discrimination within health services that should provide care and protection. Health professionals’ hostility manifested through gender and sexual discrimination, in addition to common systemic barriers such as long waiting times, lack of medical staff, and inconsistent information. These experiences often led to confrontation and embarrassment, discouraging engagement with health services. Consequently, health care for this population is shaped by a vicious cycle involving gender-based discrimination, insufficient professional training, and limited public policies to ensure rights. Nevertheless, isolated reports of positive experiences were identified, particularly in contexts where specialized services had been expanded [15].

These findings are corroborated by literature reviews on the mental health conditions of trans women and *travestis* in Brazil, which document high prevalence of major depressive disorder, anxiety disorders, suicidal ideation, suicide attempts, and substance abuse. These data indicate a significant mental health burden in this population [13,16].

Mental health outcomes reported in Brazilian studies [6,16] are consistent with international evidence. An umbrella review synthesizing 24 systematic reviews found robust evidence of adverse mental health outcomes among transgender, travesti, and nonbinary adults. Approximately half of participants reported suicidal ideation, and nearly one-third had attempted suicide at least once, rates substantially higher than those observed in the general population. The review also identified major research gaps, including limited intersectional analyses (particularly regarding race/ethnicity), scarce data on older adults and rural populations, and a lack of longitudinal research [11].

This context underscores the need to investigate life-course trajectories of TGD people and to explore non-biomedical care resources, given that structural barriers, stigma, and social exclusion restrict access to formal health services and demand non-pathologizing approaches to mental health care. From a public health perspective, understanding mental health among historically marginalized groups requires attention to how power relations, institutional discourses, and social norms shape vulnerability, access to care, and the legitimization of certain forms of life.

Although substantial research documents mental health disparities among trans and gender-diverse populations, public health scholarship has predominantly emphasized biomedical and clinical responses to these inequalities. Less attention has been given to the everyday, non-biomedical resources that individuals mobilize to sustain mental health across different life stages. Addressing this gap is essential for expanding public mental health frameworks beyond deficit-oriented models.

This marginalizing context is socially and historically structured through what Foucault conceptualizes as biopower—mechanisms through which bodies are regulated, classified, and governed [17,18]. As a regulatory norm, gender power delineates which bodies are considered socially intelligible, defining whose lives are protected and whose are rendered disposable [19]. These mechanisms operate concretely within health systems, shaping eligibility for care, institutional recognition, and exposure to avoidable psychological suffering among TGD populations.

Within dominant biomedical discourse [20], trans bodies are frequently framed through deficit, incongruence, and pathology, legitimizing practices of surveillance and medical control over access to bodily modifications and negatively impacting mental health [21]. These power regimes traverse life trajectories from adolescence into adulthood, producing cumulative exposure to violence, exclusion, and psychological distress—core elements of the social determinants of health. In the present study, these regulatory dynamics are examined not only as sources of distress but also as conditions under which participants develop artistic and playful practices to negotiate recognition and sustain mental health.

Although numerous studies document adverse mental health outcomes among trans populations, much of this literature relies on cross-sectional or convenience samples. As a result, few studies examine how barriers to mental health care change across the life course [12]. While longitudinal studies are needed to assess changes in access barriers over time, retrospective qualitative accounts provide an exploratory view of how such experiences are narrated across the life course.

Beyond temporality, life-course analysis must consider the social and symbolic processes through which subjects are constituted. Drawing on Foucault [17,18] and Butler [19,22], power is understood not only as repressive but as productive, operating ambivalently in processes of subjection and agency. Gender norms are reiteratively performed [23] across the life course, yet remain open to variation and displacement, allowing for the emergence of non-normative forms of existence. This theoretical framework guides the present study without presupposing subject passivity in relation to normative structures.

Within trajectories marked by subjection, stigma, and structural barriers to care, artistic and playful practices employed by trans and gender-diverse individuals can be understood as performative actions that facilitate the symbolic expression and emotional regulation of distressing experiences, contributing to identity affirmation, social recognition, and functioning as non-biomedical mental health care resources. This perspective allows artistic and playful practices to be interpreted as everyday performative actions through which participants rework norms, produce meaning, and construct livable identities across time.

International qualitative studies suggest that artistic expression serves as an important source of coping and agency among trans individuals. Research with trans youth in the United States has shown that artistic expression—both online and offline—is mobilized as a means of authentic self-expression, coping with distress, social connection, and autonomy, particularly among individuals experiencing multiple forms of marginalization [24]. Other qualitative studies indicate that alignment between gender identity and artistic or playful expression may support psychological well-being, highlighting the potential of expressive practices as non-biomedical mental health resources [25].

Despite these findings, existing studies often focus on specific age groups or isolated experiences, without examining how artistic and playful practices are integrated across life trajectories or how they intersect with structural conditions shaping mental health from a public health perspective. In this study, such practices are conceptualized broadly as everyday expressive activities that facilitate communication of experience and meaning making across the life course.

Beyond documenting disparities, this study advances public mental health scholarship by reframing care as a set of culturally situated, everyday practices that may function as resilience-promoting and community-based mental health resources under conditions of structural stigma.

Accordingly, the present study aims to understand, based on the narratives of trans and gender-diverse individuals, how artistic and playful resources contribute to mental health across life trajectories, considering contexts of social inequities and social determinants of health.

## 2. Materials and Methods

### 2.1. Design

The study adopted a qualitative, exploratory, cross-sectional design based on retrospective life-course narratives. A qualitative approach was chosen to facilitate an in-depth understanding of the meanings, experiences, and interpretations participants attributed to different stages of their life trajectories as recalled from the present [26]. This approach aligns with qualitative life-history and arts-based research traditions, in which analytic value derives from narrative depth, contextual richness, and interpretative coherence rather than numerical breadth.

### 2.2. Participants

The study involved four trans and gender-diverse individuals, aged 18 to 27. Three participants identified as nonbinary, and one identified as a transgender man. Sociodemographic characteristics are presented in Table 1. Inclusion criteria were: (a) aged 18 years or older; (b) self-identification as transgender and/or nonbinary; (c) engagement in artistic practices and the ability to share selected works digitally (e.g., images/files) for remote participation; (d) literacy; and (e) access to the internet and an electronic device for remote participation. Individuals who did not meet all these criteria were excluded.

The ability to share selected artworks digitally was required because the second interview stage relied on participants selecting and presenting original works within a remote format. Digital platforms also constitute contemporary spaces of artistic circulation, identity expression, and social interaction among TGD individuals. However, this criterion may limit transferability to TGD people who cannot readily digitize or share their work or who experience restricted digital access.

Consistent with qualitative methodological principles [27], although the sample size was small, the study prioritized narrative depth and interpretative richness over numerical breadth. The two-stage, extensive interviews generated dense empirical material, supporting an in-depth thematic analysis rather than statistical generalization.

### 2.3. Setting

The study was conducted entirely online via Google Meet (Google LLC, Mountain View, CA, USA) in 2022, consistent with remote data-collection procedures adopted during the COVID-19 pandemic period. Participants were instructed to attend the interviews in a private, quiet environment to minimize external interference and ensure a safe space for expression. No third parties were present during the sessions.

### 2.4. Instruments

The following instruments were used: (1) a sociodemographic questionnaire to characterize the participants; (2) a semi-structured interview guide focused on experiences with gender norms, identity recognition processes, and life trajectories; and (3) a self-interpretation guide for artistic production, through which participants selected one to three meaningful original artworks to serve as the basis for the interview.

### 2.5. Recruitment and Data Collection

Participants were recruited using snowball sampling, a strategy appropriate for reaching specific or hard-to-reach populations [28]. Initially, two TGD digital artists were identified through public profiles on social media platforms (Instagram and X) [29] and invited to participate or refer individuals within their networks who met the inclusion criteria. Recruitment continued until the final sample size was reached.

Following initial contact, participants received detailed information regarding the study objectives, procedures, and their rights. These explanations were provided in written, audio, or video format, according to participant preference. Participation was formalized through an informed consent form. Subsequently, participants completed a sociodemographic questionnaire via email.

Data collection occurred in two stages: (1) Stage 1 consisted of a semi-structured interview (60–90 min) conducted remotely (see Section 2.3), focusing on participants’ narrated life trajectories and experiences with gender norms; (2) Stage 2 consisted of a semi-structured interview (60–90 min) centered on the interpretation of participants’ own artworks, exploring the subjective, affective, and identity-related meanings attributed to their artistic productions.

All interviews were audio and video-recorded with prior authorization and transcribed verbatim. Transcriptions followed a non-naturalistic approach, prioritizing semantic content. Minor grammatical and oral adjustments were made solely to ensure readability without altering the participants’ original meanings. Potentially identifying information was removed or pseudonymized to preserve anonymity.

### 2.6. Data Analysis

Data were analyzed using Reflexive Thematic Analysis. Given the small sample size, alternative qualitative approaches such as Interpretative Phenomenological Analysis (IPA) were considered. However, IPA is primarily idiographic and oriented toward detailed case-by-case examination of individual lived experience. In contrast, the present study aimed to identify patterned meanings across participants’ narratives in relation to broader social determinants of health and life-course processes. Reflexive Thematic Analysis was therefore considered more appropriate to support a theoretically informed, pattern-based analysis consistent with the study’s public health framework [27].

The analytical process combined deductive and inductive procedures; initially, a set of a priori codes and categories was defined based on the study objectives and relevant literature. This initial coding framework functioned as a flexible and provisional guide and was iteratively revised throughout the coding stage, consistent with the reflexive orientation of thematic analysis.

Subsequently, transcripts were read in depth, coded, and reviewed, allowing themes to be actively developed through engagement with the empirical material. The analysis was conducted reflexively, considering both the meanings attributed by participants and the broader social and structural contexts shaping their narratives. Final analytical themes were constructed through the integration of these procedures, grounded in patterns of shared meaning identified across the dataset.

### 2.7. Research Team and Positionality

This qualitative study was conducted and reported in accordance with the Consolidated Criteria for Reporting Qualitative Research (COREQ) checklist [30]. Data collection was carried out through semi-structured interviews conducted by the second author, who at the time was an undergraduate psychology student with prior experience in qualitative interviewing.

There was no prior relationship between the interviewer and participants. Field notes were not produced after the interviews. Interviews were audio and video-recorded and transcribed verbatim with the assistance of transcription software (Reshape—Version 1.0.5, Reshape AI Ltd., London, UK), following a non-naturalistic transcription model. Data analysis was conducted using reflexive thematic analysis with the support of a flexible and iteratively refined coding framework.

Member checking was not performed. In line with reflexive thematic analysis, analytic rigor was supported through prolonged engagement with the dataset (two interviews per participant), iterative coding and theme refinement, systematic use of verbatim excerpts, and ongoing reflexive discussion between authors regarding interpretative decisions.

Both authors identify as cisgender individuals and are not members of the trans and gender-diverse community. We recognize that occupying a cisgender social position may entail relative privilege and may shape both interactional dynamics during data collection and interpretative perspectives during analysis. Accordingly, we explicitly attended to potential power asymmetries and to the risk of importing cisnormative assumptions into coding and theme development.

Our interest in this topic derives from prior academic engagement with gender studies, public health inequities, and the social determinants of mental health, rather than from shared identity with participants. We approached the interviews with the expectation that artistic practices would be described mainly as individual coping strategies. Participants’ accounts, however, expanded this expectation by emphasizing collective, political, and temporally transformative dimensions of artistic production. This shift prompted us to broaden the analytic framing beyond individual coping toward community-based and public health perspectives.

While the absence of shared gender identity may limit access to certain embodied dimensions of gender marginalization, we treated these limits as epistemological boundaries of the study and sought to mitigate them by centering participants’ meanings, using verbatim excerpts, and maintaining transparency about interpretative decisions.

### 2.8. Ethical Considerations

Participants provided informed consent prior to participation. Interviews were conducted remotely in a private setting chosen by participants, and no third parties were present during the sessions. Potentially identifying information was removed or pseudonymized to protect confidentiality. Participants were informed that they could pause or discontinue participation at any time without penalty.

## 3. Results

In line with reflexive thematic analysis, themes were developed from patterned meanings across the dataset rather than from uniform contributions by all participants. The prominence of individual participants therefore varies by the relevance and salience of their narrated experiences to each theme.

### 3.1. Gender Norms, Social Expectations, and Contexts of Health Inequity

This theme highlights the social determinants of mental health that shape the life trajectories of the trans and gender-diverse participants, particularly binary gender norms, heteronormativity, and structural inequalities that condition psychological distress and unequal access to care. Across childhood and adolescence, participants described early and persistent exposure to binary gender norms within family and school contexts, shaping trajectories of distress and constrained self-expression.

In their narratives, participants reported early and persistent exposure to binary gender norms, experienced as regulatory frameworks organizing family relationships, social interactions, and possibilities of social recognition. These norms were associated with fear, withdrawal, emotional suffering, and restrictions on subjective expression, particularly during childhood and adolescence.

Samy described experiences of physical and symbolic violence within the family context related to attempts to “correct” dissident gender expressions. These experiences were reported as producing withdrawal and self-censorship: “[…] my mom hit me a lot, saying things like ‘is this what you want for your life, to be a sissy?’ […] that really marked me […] and after that I became more withdrawn.”

Val reported early internalization of masculinity norms as family-imposed expectations, particularly mediated by the father, associated with emotional coercion and the naturalization of prescribed behaviors: “[…] I think there’s this issue of children internalizing how family relationships work and how gender is structured in those relationships. But in my case, it was more through imposition. I remember moments when my father was somewhat prohibitive about certain things—things that were considered ‘for girls’.”

Léo described gender binarism as particularly rigid for trans men, emphasizing that demands for conformity to normative models of masculinity function as sources of exclusion and distress: “[…] there could be more openness to understanding that binarity doesn’t have to be stereotyped […] it’s basically a stereotyped norm that binary people are expected to follow.” Nick critically framed binary gender norms as historically constructed and scientifically reductionist. Nick reported that distress intensified during puberty, when bodily changes increased social surveillance and subjective discomfort.

Overall, these findings situate TGD mental health not as an individual phenomenon, but as an outcome of structural processes of inequality, constituting a context of health inequities within which care strategies—including artistic and playful resources—are mobilized across the life course. These accounts illustrate how gender norms operate as regulatory mechanisms that structure exposure to minority stress across the life course.

### 3.2. Life Trajectories, Family Relationships and Identity Recognition Processes

This theme illustrates how identity recognition processes, shaped by unequal family and social contexts, directly impact mental health across life trajectories. Across different stages of life—from early questioning to later social affirmation—participants described identity recognition as gradual and non-linear, often shaped by shifting family dynamics and access to supportive networks.

Participants described the recognition of their gender identities as gradual, non-linear, and often silent processes, marked by fear of rejection, internal negotiation, and self-protective strategies. Family relationships emerged as ambivalent, simultaneously functioning as sources of affection and as spaces of control, misunderstanding, or delayed acceptance.

Samy also described her identity recognition process as gradual and shaped by family tensions, particularly in relation to early experiences of rejection and withdrawal. Val reported that the possibility of questioning gender norms emerged alongside reflections on sexuality, allowing greater flexibility regarding rigid masculinity expectations: “[…] realizing that it was okay for me to like boys too […] came together with realizing that it was also okay not to perform masculinity 100% of the time, not having to be a very macho man.” Léo described an initial identification as gender fluid, later understood as an adaptive strategy in response to social expectations, prior to affirming a trans masculine identity: “[…] I realized that understanding myself as gender fluid was basically me masking myself […] to meet society’s expectations of me.” Nick identified puberty as a critical turning point, during which bodily and emotional distress intensified and prompted the search for new subjective and institutional reference points and sources of support.

Across narratives, the construction of extrafamilial support networks—including friendships, affective partnerships, peer groups, and health services—was described as a key protective factor for mental health, particularly in contexts of partial or delayed family recognition. These findings underscore the relevance of community-based care as a positive social determinant of health. Identity recognition emerged as relationally constituted, shaped by available conditions of social recognition and support.

### 3.3. Artistic and Playful Resources as Strategies for Subjective Elaboration and Mental Health Promotion

This theme addresses how artistic and playful resources operate as mental health care strategies, particularly in contexts of social vulnerability, symbolic violence, and unequal access to formal health services. In adolescence and early adulthood, artistic and playful practices were described as accessible, everyday strategies for coping with distress, communicating affect, and sustaining identity negotiation—particularly when formal mental health care was experienced as limited or unsafe.

Samy described art as an embodied and continuous form of self-expression, referring to it as “living art,” through which she elaborated experiences of violence, anxiety, and identity negotiation. As she stated, art was “a way of feeling and expressing what I could not say otherwise.”

Artistic practices emerged as central resources for emotional regulation, subjective elaboration, and psychological well-being. Participants described these practices as everyday forms of care rather than ancillary activities.

Val described music as a mediated form of expression that enabled the communication of affect in abstract and protected ways, supporting emotional organization and self-regulation: “[…] it’s not exactly synesthetic […] it’s about relating a sense to an emotion […] something more abstract in that sense.” Léo reported that creating characters and narratives functioned as a privileged space for elaborating anxiety, family conflict, and psychological distress, later recognizing the projection of emotional burdens onto these characters: “[…] he [the character] is basically the one who holds my emotional load.” Nick described art as a fundamental human expression and care resource, particularly during moments of distress: “[…] when I’m not feeling well, I can just look at the painting […] think about that place and imagine it as a good place to be.”

These findings indicate that artistic and playful resources function as non-biomedical strategies for mental health promotion, expanding care repertoire in contexts marked by institutional barriers and social inequalities. All participants described artistic or playful practices as meaningful resources for emotional regulation, although the modalities and symbolic meanings varied across narratives. Artistic practices functioned as everyday technologies of care, particularly in contexts where formal services were seen as inaccessible or unsafe.

### 3.4. Artistic Processes, Temporality, and Emotional Regulation Across the Life Course

Whereas Section 3.3 focuses on the functions of artistic practices as mental health strategies, Section 3.4 emphasizes how these functions unfold temporally through processes of revisiting, transforming, and re-signifying experience across life stages.

This theme highlights how artistic resources support mental health over time, emphasizing the creative process, temporality, and the re-signification of emotional experiences. Across the life course, participants emphasized temporality: returning to earlier work, reformulating meanings, and using creation as a repeated process of emotional reorganization over time. All four participants contributed to this theme, although the emphasis on temporality varied across narratives.

Samy similarly described artistic creation as a process of re-signification over time. Earlier performances centered on themes of death and self-annihilation, which she later reformulated into collective and life-affirming actions. She also reported repeatedly revisiting and modifying previous artworks, describing this practice as a way of transforming emotional states and sustaining continuity across different moments of her life trajectory.

Participants consistently emphasized that the mental health benefits of art were primarily associated with the process of creation rather than the final product, allowing flexibility, experimentation, and tolerance of incompleteness. Val described the continuity of artistic production as reflecting the persistence of affective experiences over time, recognizing past emotional states in previously created works. Nick explicitly addressed the temporal dimension of art, describing the recurring practice of “painting over” previous works as a strategy for transforming frustration into renewed possibility: “[…] building something for the future, something that might not exist now, but that you hope will exist.”

These accounts suggest that care unfolds through iterative processes of reworking and re-signification over time rather than through singular expressive acts.

### 3.5. Representation, Artistic Production, and Collective Care in Contexts of Inequality

This theme demonstrates that artistic and playful resources extend beyond individual care, operating as mechanisms of social recognition, belonging, and collective care, which are central to public mental health among marginalized populations. In early adulthood, participants described artistic circulation and collective engagement as forms of social recognition and community-based care, strengthening belonging and future-oriented hope. Although collective engagement was more explicitly emphasized by some participants, elements of relational and community-oriented meaning were present across narratives.

Léo explicitly articulated the political dimension of artistic production, emphasizing the creation of trans, queer, and neurodivergent characters as a response to the absence of affirmative representations and as a form of community-based care. Nick described poetry as a means of engaging cisgender audiences, fostering reflection on structural violence and inequalities affecting TGD health and well-being.

Samy emphasized the collective dimension of artistic production, describing her involvement in LGBTQIA+ collectives and public performances as strategies to foster belonging, mutual support, and future-oriented hope within marginalized communities.

Across narratives, participants understood the circulation of art as situated and relational, capable of producing meaningful impacts within specific social contexts by strengthening networks of support, recognition, and collective care. Artistic circulation extended mental health support beyond the individual, reinforcing collective recognition and belonging.

## 4. Discussion

This study extends public mental health knowledge in three ways: first, by foregrounding everyday, non-biomedical practices as part of the care repertoire mobilized under structural stigma; second, by integrating these practices within a life-course framework; and third, by highlighting the relational and collective dimensions of artistic production as mental health resources.

The findings corroborate the public health literature by indicating that psychological distress among TGD people should not be interpreted as an individual phenomenon or as intrinsic to gender diversity, but rather as an effect of social determinants of health [6,8,11], particularly heteronormativity, gender binarism [19], and exclusion.

Participants’ narratives indicate that binary gender norms operate as a regulatory apparatus, in Butler’s terms [19,22], shaping life trajectories from childhood and adolescence and producing repeated experiences of invalidation, coercion, and symbolic and material violence [10]. For instance, Samy’s experience of familial punishment for gender expression illustrates regulatory power operating within domestic contexts, while her later reformulation of artistic performances exemplifies performative reworking across time. These mechanisms operate both at the microsocial level—within family and interpersonal relationships—and at the institutional level, structuring bodily, identity-related, and behavioral expectations that delimit which lives are recognizable and socially legitimate.

Considering Butler [19,22], gender emerges as a reiterated norm that produces social intelligibility at the cost of excluding bodies that do not align with a heteronormative matrix. Participants’ accounts illustrate how this matrix functions as a regime that naturalizes suffering [21], rendering gender nonconformity a legitimate target of social sanction.

These processes constitute chronic exposures to minority stress [10] and reinforce evidence linking mental health inequalities to adverse social contexts, structural discrimination, and limited access to gender-affirming care [9,12]. Thus, psychological distress emerges as a socially produced and unequally distributed phenomenon, representing a central public health concern.

The life-trajectory analysis indicates that identity recognition processes are gradual, non-linear, and strongly conditioned by the social and institutional relations available across the life course. Consistent with life-course approaches in public health, critical events—such as puberty, leaving the family home, or gaining access to community networks—function as turning points with meaningful impacts on mental health.

From a Foucauldian perspective [17,18], these moments can be understood as periods of intensified exposure to norms governing bodies, sex, and desire, requiring strategies of negotiation, silencing, or resistance. Butler [22] further suggests that recognition is not solely an internal process, but a precarious relation to social norms that grant—or withdraw—the possibility of socially recognized existence.

Family relationships were described as ambivalent, functioning simultaneously as sources of care and as sites of control and suffering. This finding aligns with national studies indicating that family rejection and symbolic violence in domestic settings constitute major barriers to mental health care for transgender people [13,15]. Here, we retain the wording used in the cited Brazilian studies, which focus primarily on trans women and travestis and do not consistently employ the umbrella term trans and gender-diverse across identity categories.

Conversely, the development of extrafamilial support networks—including peers, intimate partners, artistic collectives, and specialized services—emerged as a key protective factor. These ties expanded subjective resources, reduced social isolation, and supported identity recognition processes, consistent with evidence that social support is a central determinant of mental health for TGD populations [16].

A central contribution of this study to public health is the evidence that artistic [31] and playful practices function as non-biomedical mental health care resources across trans and gender-diverse people’s life trajectories. Unlike formal clinical interventions, these practices were mobilized in everyday, situated, and self-managed ways, supporting coping, emotional processing, and meaning making.

Music, performance, drawing, painting, and poetry writing were described as devices for emotional regulation and for articulating what may be otherwise unspeakable, enabling the organization of internal experience and the reduction in psychological distress. These findings align with studies identifying art as a source of coping, authenticity, and agency among transgender and gender-diverse youth [24,25].

From a Foucauldian standpoint [17,18], such practices can be understood as technologies of the self through which individuals produce singular forms of care, resistance, and self-governance in contexts marked by normative control, stigmatization, and institutional exclusion. In public health terms, these practices expand the repertoire of care beyond the biomedical model, particularly where access to formal services is limited, precarious, or shaped by experiences of discrimination [32]. For example, Nick’s description of repeatedly “painting over” previous works reflects an active practice of self-formation through aesthetic re-elaboration of emotional experience over time.

The findings also indicate that the beneficial effects of artistic practices on mental health are strongly associated with the creative process rather than solely with the final product (the artwork). Artistic creation was described as a continuous, non-linear, and temporally situated process, enabling revisiting, transformation, and re-signification of experiences over time.

This temporal dimension aligns with both life-course approaches in public health and Butler’s concept of performativity [23] as reiteration open to difference. The possibility of “redoing,” “restarting,” or “transforming” a work function as a practical metaphor for psychic flexibility, frustration tolerance, and reworking of suffering. In this sense, art not only expresses suffering but actively participates in the production of health by sustaining hope, continuity, and future orientation, even under persistent structural inequities.

Beyond individual care, the findings indicate that artistic production has a collective and political dimension, operating as a resource for social recognition, community belonging, and collective care [32]. The creation of characters, narratives, and works representing transgender experiences emerges as a response to invisibility and stigmatization in hegemonic social spaces. This finding is consistent with public health perspectives [33] emphasizing cultural, collective, and community-based strategies as central to mental health promotion among historically marginalized populations.

From a public health perspective, these findings highlight how non-biomedical and culturally situated care practices may mitigate mental health inequalities produced by structural stigma and barriers to formal services. Recognizing artistic and playful resources as legitimate forms of care may inform more comprehensive and equity-oriented mental health policies for trans and gender-diverse populations.

Practically, public health systems may integrate low-threshold expressive activities—such as community art workshops, peer-led creative groups, or partnerships with LGBTQIA+ collectives—as complementary mental health promotion strategies. Training initiatives for primary and community-based care providers could also incorporate recognition of non-biomedical, culturally situated practices as part of comprehensive and equity-oriented care. In the Brazilian SUS context, such initiatives could be embedded in primary health care and community mental health actions in partnership with local LGBTQIA+ collectives.

It is also important to acknowledge that the socioeconomic conditions of participants were not formally assessed beyond educational level and self-reported racial/ethnic identification. Although artistic and playful practices emerged as accessible and meaningful resources within this sample, some forms of artistic engagement—such as access to musical instruments, digital tools, or art materials—may depend on material resources that are unevenly distributed. In contexts of economic precarity, particularly among TGD populations disproportionately affected by poverty and labor exclusion, access to certain artistic modalities may be constrained. Therefore, the findings should not be interpreted as suggesting that all artistic practices are universally accessible, but rather as illustrating how such resources operated within the specific socioeconomic context of this sample. Future research should explicitly assess socioeconomic conditions and examine how material constraints shape access to different artistic modalities and their potential as mental health resources.

## 5. Conclusions

This study aimed to examine how artistic and playful resources contribute to trans and gender-diverse people’s mental health across life trajectories, considering contexts of inequities and social determinants of health. Overall, the findings indicate that the proposed objective was achieved by showing that expressive practices play a relevant role in sustaining mental health in contexts marked by stigmatization, social exclusion, and structural barriers to care.

Regarding binary gender norms, repeated exposure to heteronormative regimes was associated with experiences of psychological distress—such as anguish and sadness—across the life course. These findings reinforce the interpretation that such experiences should not be read as individual phenomena, but as effects of social determinants of health operating from childhood and extending through family, school, and institutional contexts.

Regarding performativity, the findings indicate that processes of identity recognition and affirmation occurred gradually and non-linearly and were supported, at different points, by extrafamilial support networks. The presence of meaningful social ties—including peers, collectives, and relationships mediated through art—was associated with expanded subjective resources, the construction of future perspectives, and the production of meanings that supported life continuity and coping with suffering.

Art, integrated into these processes, emerged as a central non-biomedical care resource. Artistic practices were mobilized as strategies for emotional processing, subjective expression, belonging, and agency, enabling participants to build symbolic and social spaces of existence in contexts where their identities were frequently delegitimized. Thus, art did not function merely as an expressive medium but as an active resource to produce mental health across life trajectories.

Given the exploratory nature of the study, several limitations should be acknowledged. The volume and density of the empirical material generated a dense *corpus,* requiring analytic prioritization. Given the scope of a single article, it was not possible to exhaustively explore all narrative layers, symbolic dimensions, and intra-participant variations contained in the dataset. The analysis therefore focused on cross-cutting patterns of meaning aligned with the study’s public health framework, rather than conducting detailed case-by-case or multimodal analysis of each artistic production.

In addition, the sociopolitical and public health context in which the research was conducted—including public discourse delegitimizing LGBTQIA+ identities during the administration of President Jair Messias Bolsonaro (2019–2022), as well as a period marked by contested risk communication and disputes over the implementation of public health measures during the COVID-19 pandemic—may have shaped participants’ reported experiences and should be considered when interpreting the findings. The remote interview format, adopted in accordance with pandemic-related public health recommendations, may also have affected interactional spontaneity and the expression of more sensitive experiences. Participants were young adults within a specific Brazilian sociopolitical context, and findings may not directly apply to older trans and gender-diverse individuals, rural populations, or contexts with different health system structures.

On the other hand, the use of the Consolidated Criteria for Reporting Qualitative Research (COREQ) enhanced the reporting quality, together with the nature of qualitative inquiry as oriented toward meanings, worldviews, and values rather than quantification or statistical generalization.

Despite these limitations, the study contributes to public health by reinforcing the relevance of approaches that recognize cultural, artistic, and expressive practices as legitimate components of mental health care. In the Brazilian context, characterized by regional inequalities and weaknesses in implementing policies specific to trans and gender-diverse populations, valuing these resources may support more equitable, culturally sensitive care strategies aligned with the principles of comprehensiveness and equity of the Brazilian Unified Health System (SUS).

Given the cross-sectional and retrospective design of the present study, future longitudinal qualitative and quantitative research is needed to examine how artistic and non-biomedical mental health resources evolve over time and across changing sociopolitical contexts.

Recognizing artistic and playful practices as legitimate forms of care expands public mental health beyond clinic-centered responses and supports equity-oriented strategies aligned with the principles of comprehensiveness in the Brazilian Unified Health System (SUS). Future research should include larger and more socioeconomically diverse qualitative samples, participatory and community-based designs, and longitudinal approaches to examine how expressive practices evolve across changing social contexts. These findings are exploratory and illustrative, intended to inform conceptual and public health discussions rather than to estimate population prevalence.

## Figures and Tables

**Table 1:** Sociodemographic characterization of participants (*n* = 4).

Name	Age (Years)	Gender Identity	Race/ Ethnicity	Educational Level	Main Artistic Practice
Samy	27	Nonbinary	Black	Higher Education completed	Digital visual arts, performance, text writing
Val	20	Nonbinary	White	Incomplete Higher Education	Music, composition, plays cello and guitar
Léo	19	Nonbinary	White	Incomplete Higher Education	Landscape painting, acrylics and oils, writes poems, handicrafts
Nick	18	Transgender man	Black	Incomplete Higher Education	Digital art, comics, writing stories and texts

## Data Availability

The data supporting the findings of this study are available from the corresponding author upon reasonable request for the purpose of peer review. However, the data cannot be made publicly available due to ethical considerations and the need to protect the confidentiality and privacy of the study participants.

## Abbreviations

The following abbreviations are used in this manuscript:

CAAE	Certificate of Presentation of Ethical Assessment
TGD	Trans and Gender-Diverse
SUS	Brazilian Unified Health System
